# Bilateral NIRS measurements of muscle mitochondrial capacity: Feasibility and repeatability

**DOI:** 10.14814/phy2.14826

**Published:** 2021-05-04

**Authors:** Rewais Hanna, Jigar Gosalia, Alaina Demalis, Zachary Hobson, Kevin K. McCully, Brian A. Irving, Swapan Mookerjee, Giampietro L. Vairo, David N. Proctor

**Affiliations:** ^1^ Penn State University University Park PA USA; ^2^ University of Georgia Athens GA USA; ^3^ Louisiana State University Baton Rouge LA USA; ^4^ Bloomsburg University Bloomsburg PA USA

**Keywords:** electrical stimulation, ischemia, mitochondria, near infrared spectroscopy, oxidative capacity

## Abstract

**Background:**

Non‐invasive determination of mitochondrial capacity via near infrared spectroscopy (NIRS) typically involves voluntary exercise of a single muscle group followed by as many as 26 brief ischemic cuff occlusions to determine a single recovery rate constant (*k*).

**Purpose:**

To determine the within‐ and between‐visit repeatability of a shortened bilateral NIRS protocol, and to establish the feasibility of hamstring *k* measurements.

**Methods:**

Sixteen young (eight women, eight men; 22 ± 3 years) active adults underwent a bilateral electrical stimulation protocol in which multiple (*n* = 4) measurements of *k* for the vastus lateralis (VL) and medial hamstring (MH) muscles were determined on two visits. Repeatability (CV% and intraclass correlations, ICC) and equivalency across visits were assessed for both muscles.

**Results:**

Mean *k* values in the VL were consistent with published values and within‐visit ICCs were moderately high for both muscles in both sexes. In men, average *k* values on visit 2 were within 1% (VL muscle) and 5% (MH muscle) of the values on visit 1 (all *p* > 0.78). In women, average *k* values were 10%–15% lower on visit 2 (*p* = 0.01 and *p* = 0.15 for MH and VL) with the largest between‐visit differences in a subset of participants with the most days between visits.

**Conclusions:**

This bilateral NIRS protocol is time efficient and provides valid estimates of *k* in both sexes and muscle groups with acceptable within‐visit repeatability. Lower than expected between‐visit repeatability in some participants reinforces the need for further investigation of this newly developed protocol to identify and control for experimental and behavioral sources of variation.

## Graphical abstract

We developed and tested the reliability of a novel bilateral near infrared spectroscopy protocol to estimate mitochondrial capacity (*k*) in the vastus lateralis and hamstring muscles of active younger men and women. Within‐visit intraclass correlations were moderately high, but between‐visit repeatability of *k* in these muscles was lower than expected in a subset of women. Bilateral estimates of *k* are feasible and time efficient even in the hamstring muscles, but further studies employing this protocol will be needed to identify sources of variation between days.

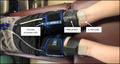

## 1 INTRODUCTION

Near infrared spectroscopy (NIRS) is a non‐invasive method used to measure changes in oxygenated and deoxygenated hemoglobin/myoglobin in various tissues, including skeletal muscle (Willingham & McCully, 2017). Hamaoka et al. (2011) and later Ryan et al. (2013) developed protocols for estimating the mitochondrial (oxidative) capacity of individual skeletal muscles that utilize NIRS to measure the rate of recovery of muscle oxygen consumption ($\dot{V}O_{2}$) following a short bout of muscle contraction. The recovery time constant (*k*) derived from the protocol of Ryan, Southern, et al. (2013), which utilizes electrical stimulation to activate muscle mitochondrial respiration, showed good agreement with measurements of phosphocreatine (PCr) recovery rates (^31^P‐NMR) and mitochondrial respiration rates from muscle biopsies (Ryan, Southern, et al., 2013, 2014, 2014). This NIRS‐based in vivo approach has also revealed relative differences in muscle mitochondrial capacity across various athlete and patient populations (Adami & Rossiter, 2018), further supporting its validity. This non‐invasive, relatively low‐cost technique is well suited to monitoring changes in muscle‐specific aerobic adaptations over time and thus offers considerable promise in evaluating the effectiveness of different interventions (exercise training, rehabilitation, medications, etc.) in research and clinical settings (Jeffries et al., 2018; Polley et al., 2016; Ryan et al., 2013).

Despite its advantages, there are limitations associated with the application of NIRS for determining muscle mitochondrial capacity. One limitation is the need to use as many as 26 ischemic cuff inflations to obtain a single value of *k* (Sumner et al., 2020). With a recommended two to three trials per muscle, per study visit to obtain a reliable estimate of mitochondrial capacity (Adami & Rossiter, 2018; Blankstein, 2011), this method is time consuming and can be uncomfortable for some subjects. An abbreviated test protocol using six repeated post exercise cuff occlusions was recently developed by Sumner et al. (2020) in an attempt to address this limitation. They found that recovery rate (*k*) constants were similar between the six‐ and 22‐cuff protocols in the upper‐limb muscles (biceps, forearm) of healthy young subjects while requiring fewer occlusion periods and less total testing time. As in several previous studies, they also used low‐frequency electrical twitch stimulation to better control the intensity and area of muscle fiber recruitment (vs. voluntary contractions with ergometers or stretch bands; Willingham & McCully, 2017).

In the present study, we adapted the six‐occlusion cuff, electrical stimulation method of estimating muscle mitochondrial capacity to the muscles acting upon the knee joint (vastus lateralis [VL] and medial hamstring [MH], which comprises the semitendinosus and semimembranosus muscles). Evaluating mitochondrial capacity in lower extremity muscles is important given their role in posture and locomotion and the chronic impact of cardiovascular and metabolic disorders on leg muscle oxygen delivery and mitochondrial function (Hart et al., 2018). The need for time efficient NIRS estimates of mitochondrial capacity is especially important for participant comfort because lower extremity muscles require higher cuff inflation pressures to adequately occlude blood flow vs. that of the upper limb (Hunt et al., 2016; Loenneke et al., 2015). NIRS‐based assessments of VL and MH muscle mitochondrial capacity also have the potential to identify physiological factors contributing to lower extremity musculoskeletal injury and evaluate the effectiveness of related therapeutic interventions to restore function. Our interest in determining the feasibility of this method for measuring mitochondrial capacity in the hamstring muscles stems from the fact that this is a frequently injured muscle group and a common site for tissue harvest in patients undergoing surgical Anterior cruciate ligament (ACL)‐reconstruction, particularly among physically active young adults.

Consequently, we developed a bilateral, electrical stimulation protocol in which the mitochondrial capacity of both VL muscles (supine position) or both MH muscles (prone position) can be determined at the same time, all within a single study visit. We hypothesized that this bilateral protocol would provide time‐efficient estimates of muscle mitochondrial capacity that are repeatable for both of these muscle groups in young physically active men and women, both within‐ and between‐testing days. We further hypothesized that in healthy active younger individuals, the mitochondrial capacity of these two knee joint muscles would be similar across limbs (i.e., right to left).

## 2 METHODS

### 2.1 Subjects

Men and women between 18 and 35 years of age from The Pennsylvania State University Park Campus were recruited for this study. Volunteers were excluded from participating if they were smokers, were taking any cardiovascular medications, had a body mass index >35 or had a prior history of major lower‐limb injuries. Six volunteers with adipose tissue at the NIRS probe sites (VL and MH muscles) that exceeded 2.0 cm, which is the estimated signal penetration limit of the Portamon NIRS device (Artinis Medical Systems; Adami & Rossiter, 2018; Blankstein, 2011), were also excluded from participating during the screening visit (discussed below). This study was approved by the University's Institutional Review Board and all subjects provided written, informed consent prior to participation.

### 2.2 Study timeline

Each subject completed an initial screening and familiarization visit (session 1) to measure adipose tissue thickness (ATT), to assess their tolerance to bilateral thigh occlusion and electrical stimulation, and to determine the appropriate stimulation intensity to adequately increase the metabolic rate of the muscle. Bilateral NIRS mitochondrial capacity testing was completed on two subsequent experimental visits (sessions 2 and 3) to determine within‐ and between‐day reproducibility. A minimum of 3 days separated the screening visit from the first experimental visit, and a minimum of 24 h separated the two experimental visits. The mean number of days separating the two experimental study visits was 11.2 ± 10.0 (range: 1–32 days).

### 2.3 Screening and familiarization session procedures

During session 1, subjects completed the informed consent process, had their height and mass measured, and filled out a general health screening form and physical activity questionnaire (IPAQ short form; Lee et al., 2011). In subjects meeting inclusion criteria, these procedures were followed by measurements of ATT over the NIRS probe placement sites (VL and MH muscles). For the initial subjects (prior to January 2020) ATT was measured using a Lange skinfold caliper (longitudinal folds). For subsequent subjects, ultrasonography was used (Philips Ultrasound). Subjects with ATT ≤2.0 cm in the VL and MH muscles measured via either method (eight women and eight men), remained eligible for the study.

**FIGURE 1:**
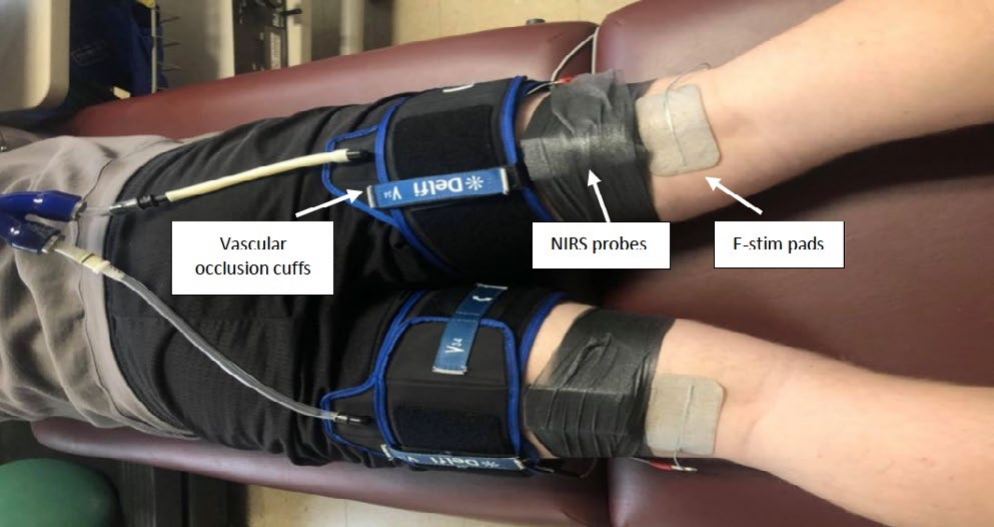
Instrument placement for the bilateral NIRS protocol (prone position for the MH muscles shown here). Upper electrical stimulation pads are beneath both occlusion cuffs. MH, medial hamstring; NIRS, near infrared spectroscopy

Each subject was then asked to lie on a padded examination table while being instrumented with upper‐thigh cuffs (tapered 4.5 × 34 inches; Delfi Medical Innovations) connected to a Hokanson large capacity reservoir inflator (Hokanson E20, D.E. Hokanson, Inc) and high‐capacity air source (SpeedAire, 11 Gallon). These components enabled rapid, simultaneous inflation of both thigh cuffs (~1 s). This was followed by a short (~1 min) simultaneous inflation of both cuffs to ensure the subject could tolerate the 250‐mmHg occlusion pressure (Loenneke et al., 2015). Next, NIRS probes (Artinis PortaMon) were placed 2/5th of the distance between the greater trochanter to the lateral patella and wrapped with opaque vet wrap (to prevent ambient light contamination) for continuous monitoring of oxyhemoglobin and deoxyhemoglobin in the VL muscle. The 2/5th proximodistal thigh length (1) consistently delineated the VL muscle as confirmed by visual inspection and palpation during isometric contraction against manual resistance (confirmed by a certified athletic trainer during pilot testing); (2) provided sufficient space for the electrical stimulation pads and upper‐thigh occlusion cuff; and (3) standardized the NIRS probe placement for between‐leg testing. Two electrical stimulation pads (Chattanooga Dura‐Stick Electrodes; DJO LLC) were then placed adjacent to each NIRS probe and connected to an electrical stimulation unit (TheraTouch Electrotherapy EX4; Richmar). Straps were then placed around each lower leg to minimize movement artefact during stimulation. Following the instrumentation setup (as shown in Figure 1), the stimulation intensity (biphasic setting, 6 Hz, 10 pulses/sec) of one VL muscle was gradually increased until a vigorous, yet tolerable muscle contraction was elicited (Ryan et al., 2013). This electrical stimulation check was also performed to ensure that metabolic rate was sufficiently increased at the final stimulation setting chosen (i.e., >three‐fold increase as determined by visual inspection of the slope of the oxyhemoglobin signal immediately post‐stimulation) for the NIRS mitochondrial capacity test to succeed. This sequence (i.e., electrical stimulation check and NIRS metabolic rate check) was then repeated for the opposite VL muscle. The subject was then assisted into the prone position for identical procedures to determine the electrical stimulation parameters for the MH muscles. For these muscles, the NIRS probes were placed approximately 2/5th of the distance between the posterior ischial tuberosity and the medial patella. This standardized distance ensured that the NIRS probes were placed over the MH muscles (semitendinosus and semimembranosus; confirmed with the assistance of a certified athletic trainer) while providing sufficient space for the electrical stimulation pads and upper‐thigh occlusion cuff. NIRS probe position was marked with indelible ink along with photos to increase accuracy for positioning during the subsequent experimental visits.

### 2.4 Mitochondrial capacity testing

Eligible subjects returned to the laboratory for two experimental sessions, to determine VL and MH mitochondrial capacity and reproducibility, using the bilateral, 6‐cuff occlusion method. Subjects were asked to avoid strenuous activity at least 24 h before each session. After instrumentation (Figure 1), electrical stimulation intensity was returned to the same levels as the familiarization session and adjusted as needed to ensure adequate contraction intensity and NIRS oxygen desaturation. Following these electrical stimulation checks, subjects rested for 5 min to allow NIRS signals to return to baseline conditions.

**FIGURE 2:**
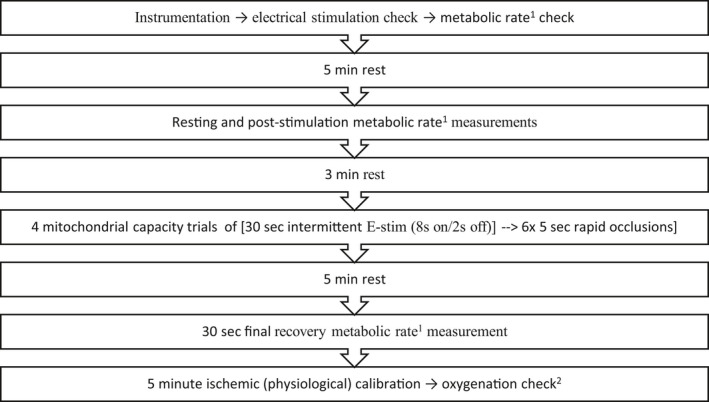
Bilateral NIRS protocol. This sequence of steps was performed first with the subject in the supine position (VL muscles), then repeated with the subject in the prone position (MH muscles). ^1^Determined from the slope of the % oxyhemoglobin saturation change during temporary cuff inflation. ^2^checked to confirm that % oxyhemoglobin saturation did not drop below 50% of the subject's physiological range for each muscle during the preceding mitochondrial capacity trials. MH, medial hamstring; NIRS, near infrared spectroscopy; VL, vastus lateralis

The mitochondrial capacity protocol was similar to the protocol developed by Sumner et al. (2020) but adapted for use in both legs. The bilateral protocol sequence, shown in Figure 2, involved four separate trials of 30 s of electrical stimulation (three cycles of 8 s stim/2 s off) followed by 6 × 5 s cuff inflation/5 s cuff deflation cycles. The rapid inflation cuff sequence was controlled using a customized LabView program. One data set (i.e., four replicate measurements) was obtained for each limb, each muscle (VL or MH), and on each day. This protocol measured resting and post‐stimulus metabolic rates to confirm adequate increases in muscle metabolism with stimulation. These measurements were obtained before the sequence of cuff inflation/deflation cycles (resting), and after this 4‐set sequence (post stimulation).

A physiological calibration of both NIRS probes was obtained after the final stimulation. This calibration identifies the difference between the minimum (nadir during a 5‐min occlusion) and maximum (post‐cuff release reactive hyperemia) oxyhemoglobin signals. Oxyhemoglobin values during the mitochondrial capacity test were checked following the physiological calibration to ensure that muscle oxygen supply did not become limiting to maximal oxidative capacity (Adami & Rossiter, 2018), i.e., not below 50% of the subject's physiological % oxyhemoglobin range for that muscle. This full protocol was then repeated for the MH muscles with the subject in the prone position.

### 2.5 NIRS measurements and analysis

Continuous wavelength NIRS probes (Artinis Portamon) with wavelengths of 765 and 854 nm were used to measure oxyhemoglobin and deoxyhemoglobin, respectively. This device has three channels with separation distances of 30, 35, and 40 mm. Data were collected via Bluetooth at 10 Hz using the Oxysoft software (Artinis Medical Systems).

**FIGURE 3:**
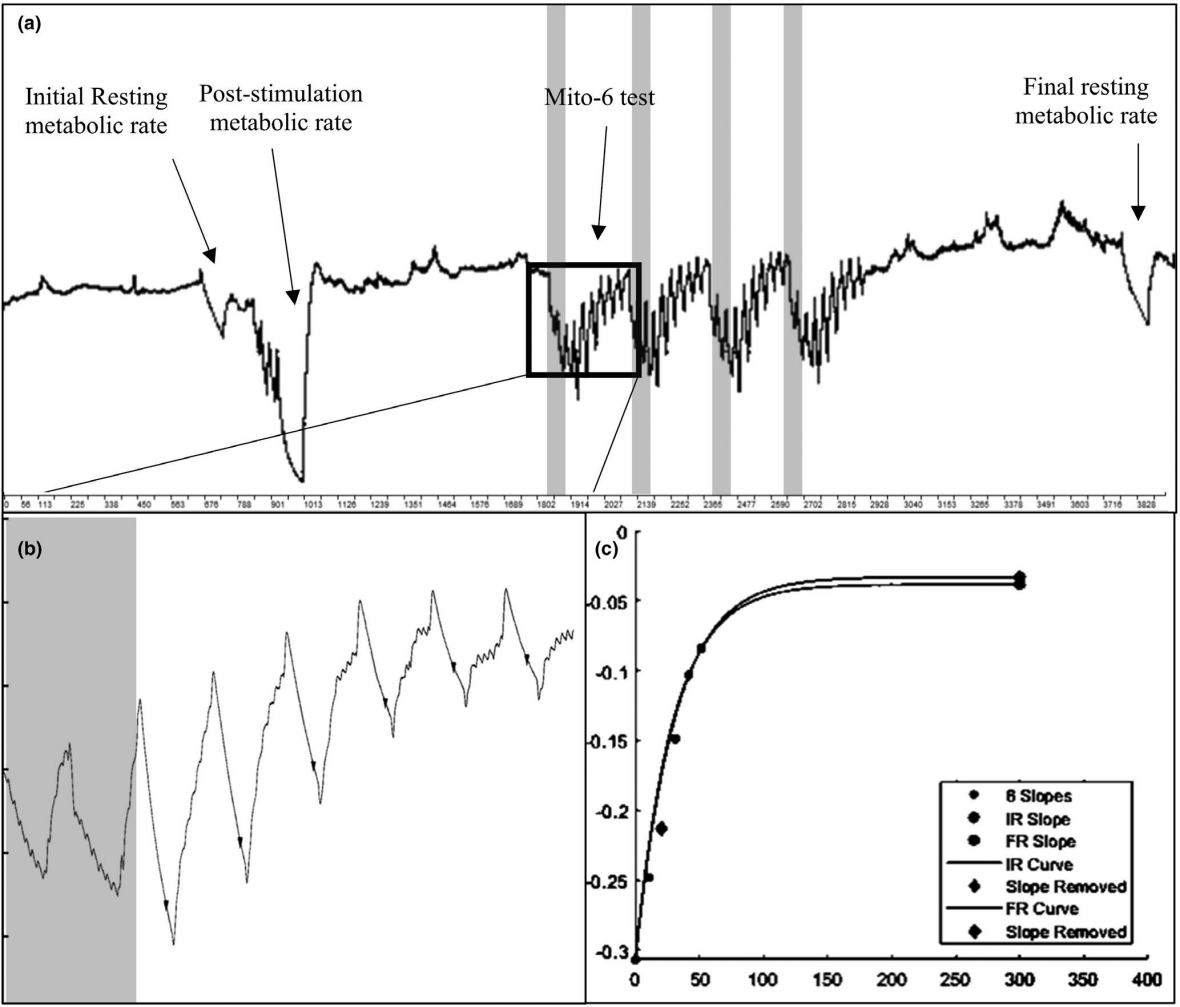
Data recordings and analysis of muscle mitochondrial capacity (*k*) for a representative subject (recordings are from one MH muscle). (a) Changes in percent oxyhemoglobin saturation during each step of the NIRS protocol. Shaded bars indicate electrical stimulation. (b) Oxyhemoglobin saturation recovery slopes for one Mito‐6 test immediately following intermittent stimulation (8 s on/2 s off). The arrows above each slope indicate the points used to calculate the NIRS recovery rate constant (*k*) in panel C. Shaded area indicates electrical stimulation. (c) Oxyhemoglobin saturation recovery slopes (i.e., six recovery values) fit onto a monoexponential curve with respect to final resting metabolic rate. The *Y* axis represents the slope, and the *X* axis represents time (s). Time to half recovery to the final resting metabolic rate is the NIRS recovery rate constant (*k*) for each trial. MH, medial hamstring; NIRS, near infrared spectroscopy

The NIRS‐derived data were exported and analyzed in MATLAB (The MathWorks, Inc.) using a proprietary system that produces the mitochondrial capacity recovery time constants (McCully & Ryan, 2017). Figure 3 shows the % oxyhemoglobin saturation changes during a bilateral 6 cuff occlusion test of the MH muscle in a representative subject.

### 2.6 Statistical analysis

Statistical analyses were performed using SPSS version 26.0 (IBM Corporation). Intraclass coefficients (ICC (2, *K)*; Koo & Li, 2016) and coefficients of variation (CV) were used to determine between‐ and within‐day variability. Equivalency tests were used to determine agreement between days (Lakens, 2017). Intra‐rater reliability, which was evaluated by having a single “blinded” rater repeat the recovery slope point selection in MATLAB on three occasions, was also evaluated using ICC and CV. Comparison of mean values between‐days and between‐limbs were analyzed using two‐sided paired *t*‐tests. A two‐sample *t*‐test assessed sex differences in mitochondrial capacity estimates for each muscle separately. An ANOVA (multi‐factor, fixed effects) was performed with factors (sex, muscle group, limb) and covariate (IPAQ score) to determine if any of these factors significantly influenced the average *k* value obtained across the two study visits. All data are presented as mean ± SD. Statistical significance was accepted when *p* < 0.05. A post‐hoc power analysis was completed for sex comparisons of mitochondrial capacity in both the VL and MH muscles (VL: *α* = 0.05, Cohen's *d* = 0.61, 1 − *β* = 0.51; MH: *α* = 0.05, Cohen's *d* = 0.50, 1 − *β* = 0.40).

## 3 RESULTS

Physical characteristics of the 16 subjects (eight men, eight women) who completed all testing sessions are presented in Table 1. Men were taller and heavier than women were, but ATT over both muscles, estimated by skinfolds or ultrasound, were similar between sexes. Self‐reported physical activity levels (IPAQ, recent weekly) were higher in women compared to men (*p* = 0.04).

**TABLE 1:** Subject characteristics

	Men	Women	*p* value
Subjects no.	8	8	
Age (years)	23.9 ± 3.6	21.3 ± 1.5	0.009
Height (m)	1.78 ± 0.13	1.66 ± 0.06	0.001
Mass (kg)	74.6 ± 18.6	57.8 ± 7.9	0.004
BMI (m^2^/kg)	23.3 ± 3.1	21.0 ± 1.9	0.052
VL ATT (mm) skinfold caliper	13.2 ± 2.4 (n=4)	14.2 ± 1.3 (n=4)	0.48
VL ATT (mm) ultrasound	12.9 ± 2.0 (n=4)	13.3 ± 1.8 (n=4)	0.82
MH ATT (mm) skinfold caliper	15.2 ± 1.5 (n=4)	15.7 ± 0.5 (n=4)	0.51
MH ATT (mm) ultrasound	13.8 ± 2.4 (n=4)	14.2 ± 1.6 (n=4)	0.74
IPAQ (METS/week)	2901 ± 1358	4217 ± 991	0.04

Values are means ± SD.

Abbreviations: ATT, adipose tissue thickness; BMI, body mass index; IPAQ, International Physical Activity Questionnaire‐short form; MH, medial hamstring; VL, vastus lateralis.

### 3.1 Feasibility of the bilateral NIRS protocol

**FIGURE 4:**
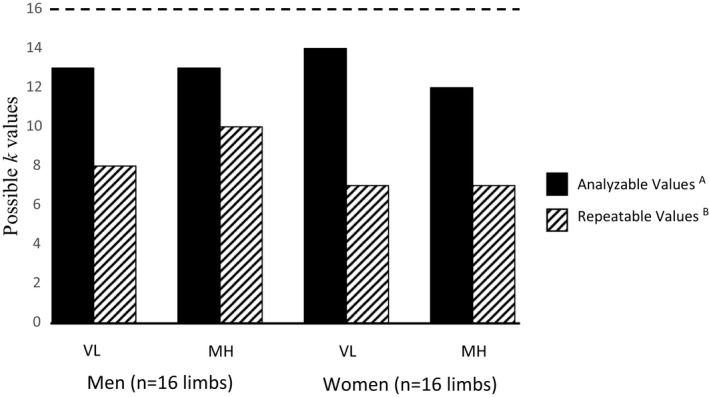
The total number of analyzable (based on monoexponential curve fit) and repeatable (based on between‐visit SD) *k* values for women and men. Dashed line indicates the maximum possible number of subjects with analyzable and repeatable rate constant measurements (i.e., *n* = 16 limbs per muscle per sex). (A) Determined by visual inspection of MATLAB‐derived monoexponential curve fit. (B) Indicates the number of trials for which the average recovery rate constant *k* was less than 1 SD unit difference between the two visits

As indicated in the methods, each subject underwent a total of 4 cycles/replicates of bilateral muscle stimulation followed by repeated bilateral cuff occlusions for each muscle on both study visits. This resulted in 16 possible rate constants for the VL muscle and 16 for the MH muscle in each subject (4 replicates × 2 limbs × 2 visits), all within two study visits each lasting approximately 2 h. Successful mitochondrial recovery curves, defined as data that fit a mono‐exponential curve (as determined by an experienced NIRS investigator and co‐author) with at least a three‐fold increase in metabolic rate (defined in Section 2), were identified in 81% of all VL muscle replicates (208 out of 256) and in 71% of all MH muscle replicates (183 out of 256) across the 16 subjects (Figure 4). Across both study visits and both limbs, all 16 subjects had an average of 12.2 ± 2.9 successful measurements out of 16 possible NIRS recovery rate constant slopes for each muscle group.

Sex differences in the repeatability of rate constant (*k*) measurements between the two visits were observed in both muscle groups; thus, most results below are presented separately for women and men.

### 3.2 Within‐visit repeatability of rate constant values

Table 2 provides the coefficient of variation and intraclass correlations (ICC) for both sexes and muscle groups. In men, within‐visit CVs ranged from 5.0% to 25.0% for both muscles with a median of 13.5% and ICC averaged 0.82. Repeatability was slightly better in the VL muscle. In women, within‐visit CVs ranged from 5.0% to 35.0% with a median of 13.0% and ICC averaged 0.86, with repeatability slightly better in the VL muscle.

**TABLE 2:** Reliability of the Bilateral NIRS Protocol

	CV (%)	ICC	*t*‐test
VL			
Intra‐rater	4.6 ± 2.5	0.98	
Men			
Within‐day	13.3 ± 5.4	0.82	
Between‐day	10.8 ± 8.1	0.92	0.72
Between‐limb	7.6 ± 6.0		
Women			
Within‐day	13.0 ± 6.2	0.86	
Between‐day	13.6 ± 9.1	0.81	0.17
Between‐limb	7.4 ± 6.8		
MH			
Intra‐rater	4.2 ± 3.3	0.97	
Men			
Within‐day	14.7 ± 5.9	0.80	
Between‐day	8.4 ± 6.4	0.87	0.57
Between‐limb	8.6 ± 6.7		
Women			
Within‐day	14.5 ± 7.2	0.82	
Between‐day	10.0 ± 6.8	0.94	0.02
Between‐limb	7.8 ± 7.1		

Values for CV are presented as means ± SD. *p*‐values are reported for *t*‐tests to test differences for average NIRS *k* values between days.

Abbreviations: CV, coefficient of variation; ICC, intra‐class correlation coefficient; MH, medial hamstring; VL, vastus lateralis.

### 3.3 Between‐visit comparisons of average rate constants

**FIGURE 5:**
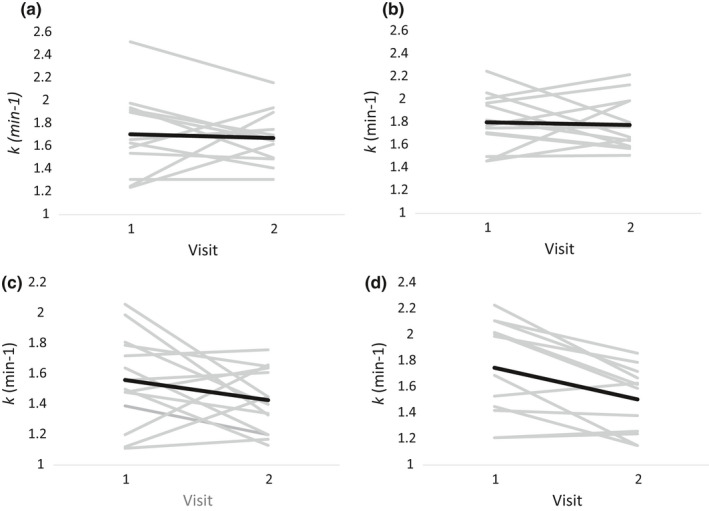
Individual NIRS‐derived *k* values between visits in men and women. Light solid lines indicate individual differences in *k* (min^−1^) between visits. Dark solid lines indicate the mean change in *k* from visit 1 to visit 2 in each muscle in each sex. (a) Men's vastus lateralis muscle (*n* = 13). (b) Men's medial hamstring muscle (*n* = 13). (c) Women's vastus lateralis muscle (*n* = 14). (d) Women's medial hamstring muscle (*n* = 12). NIRS, near infrared spectroscopy

**FIGURE 6:**
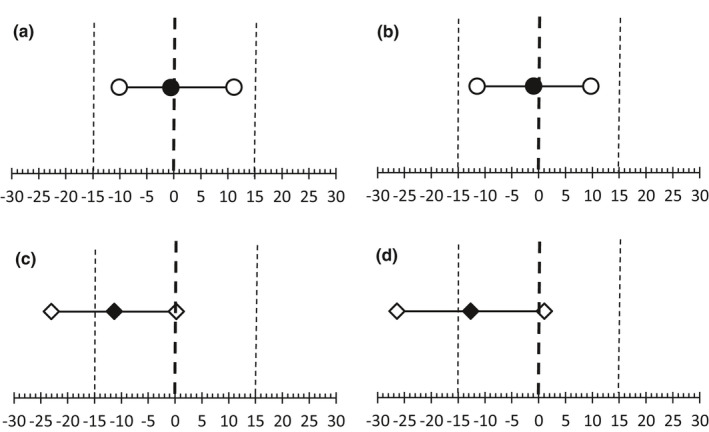
Equivalency test plots comparing the average *k* values measured on visit 2 to visit 1. The dark symbols indicate the mean difference between measurements on visits 1 and 2. The open symbols indicate the 90% confidence intervals. The *X* axis represents the difference in mitochondrial rate constants expressed as a percent (%). Vertical bold dotted lines indicate zero difference between values on visits 1 and 2. The vertical thin dotted lines indicate a difference in rate constant of 15% between visits 1 and 2. (a) Comparison of the VL muscle in men (circles). (b) Comparison of the MH muscle in men (circles). (c) Comparison of the VL muscle in women (diamonds). (d) Comparison of the MH muscle in women (diamonds). VL, vastus lateralis

In men, between‐visit CVs ranged from 0% to 29.2% with a median of 8.6% and the average *k* values for each limb on visit 2 were within 1% (VL muscle) and 5% (MH muscle) of the average values on visit 1 (*p* > 0.78 for all comparisons). However, repeatability across visits for individual subjects was quite variable (see Figure 5 showing *individual* means on both visits). In women, between‐visit CVs ranged from 1.6% to 28.1% with a median of 10.4% and the average *k* values for each limb on visit 2 were lower for both muscles compared to visit 1 and reached significance in the MH muscle (*p* = 0.01) but not the VL muscle (*p* = 0.15), with three of eight subjects showing ~20%–30% lower values on day 2 (Figures 5 and 6).

The extent to which between‐visit differences in average rate constants were considered "repeatable" (i.e., no statistical difference between days) are shown in Figure 4. Equivalency testing (Figure 6) showed that *k* values for both VL and MH muscles between days in men was statistically equivalent and not different. However, equivalency testing revealed that *k* values were not statistically equivalent for the same analysis in women. These conclusions were based on hypothesis testing in which differences in *k* values between days were not different from zero and the 90% confidence interval for the mean difference was within ±15% of the mean value.

### 3.4 Muscle group, sex, and limb comparisons of average rate constants

**FIGURE 7:**
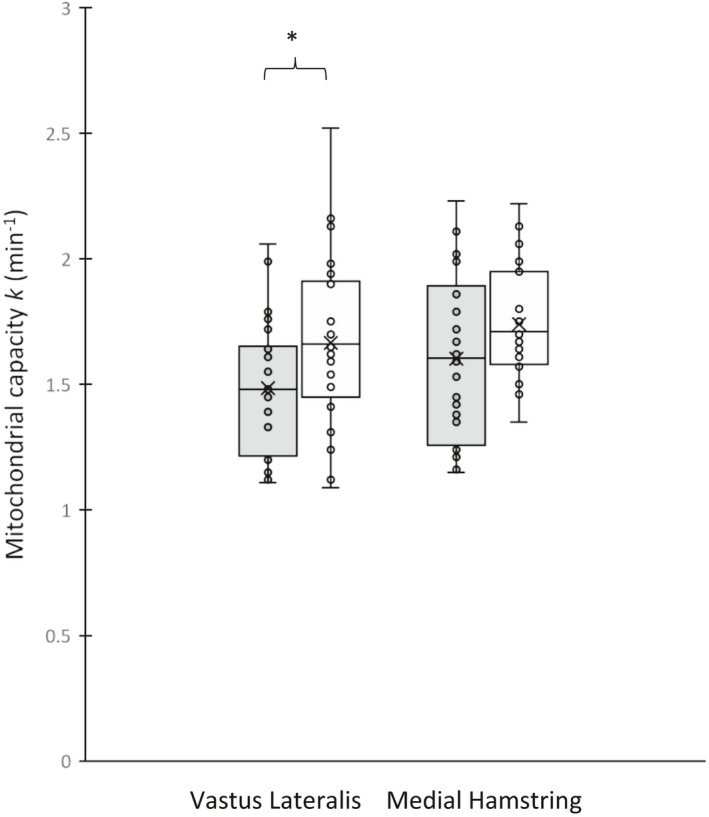
Average NIRS derived recovery constants between sexes in both muscle groups. The darker shaded bar represent the mean and individual rate constant values in women. The lighter shaded bar represent the mean and individual rate constant values in men. The average for each group is represented by the line in the box plot. The whiskers represent the range of values for each muscle in men and women. NIRS, near infrared spectroscopy. *Indicates *p* < 0.05

The average *k* values across visits for both muscles are displayed in Figure 7. Average *k* values were higher in the MH than in the VL muscle; this muscle group difference was observed during both study visits in both limbs. Average *k* values were also higher in men than in women in both muscle groups and in both limbs. There were no right‐to‐left limb differences in *k* for either muscle in either sex (all *p* > 0.10; data not shown).

An ANOVA (multi‐factor, fixed effects) was performed to determine which factors (sex, muscle group, limb) explained significant variance in the average *k* value obtained across the two study visits. This analysis showed that sex was a significant determinant of *k* (*p* = 0.012), but muscle group (*p* = 0.07), physical activity levels (*p* = 0.76) and limb (*p* = 0.91) were not.

### 3.5 Intra‐rater repeatability of rate constant curve fitting

Repeatability of NIRS recovery curve fitting by one investigator blinded to subject identity was quite high for the VL muscles (CV% = 4.6 ± 2.5, ICC = 0.98) and for the MH muscles (CV% = 4.2 ± 3.3%, ICC = 0.97).

## 4 DISCUSSION

The primary purpose of the present study was to develop, and assess the reproducibility of, a bilateral NIRS‐based protocol for measuring muscle mitochondrial capacity in the lower extremity (i.e., knee joint) muscles. The results indicate that while this protocol provides time efficient and physiologically valid estimates of mitochondrial capacity in these muscle groups with acceptable repeatability on a single study visit, there was lower than expected between‐visit repeatability of these estimates in some of our female participants for undetermined reasons. Below we discuss the feasibility and reproducibility of this protocol for both muscle groups tested, compare the derived rate constants (*k*) to the literature and between muscle groups and limbs, and provide recommendations to improve the between‐visit reproducibility of this novel bilateral protocol.

### 4.1 Feasibility of the bilateral NIRS protocol

To the best of our knowledge, this present study is the first to apply the NIRS repeated occlusions mitochondrial capacity method to both lower limbs simultaneously. Bilateral surface stimulation coupled with an automated six repeated occlusion cuff protocol resulted in a large number of muscle $\dot{V}O_{2}$ recovery trials and *k* value estimates while keeping total occlusion time and participant discomfort to a minimum. The average number of successful NIRS mitochondrial capacity trials (i.e., trials with a monoexponential $\dot{V}O_{2}$ recovery curve fit and a three‐fold or greater increase in muscle metabolic rate) in these subjects was 13.0 and 11.4 (out of 16 possible) for the VL and MH muscles, respectively. This means that for four possible replications of the *k* value for each muscle and limb (i.e., left hamstring) on a given visit, an average of three replicates were available for analysis. The present findings, along with those of Sumner et al. in upper extremity muscles (Sumner et al., 2020) support the feasibility of using partial recovery curves to estimate muscle mitochondrial capacity with NIRS in healthy active younger adults.

### 4.2 Repeatability of the bilateral NIRS protocol

In men, within‐visit CVs ranged from 5.0% to 25.0% (median = 13.5%), with slightly lower variance across the four replicates in the VL compared to MH muscles (Table 2). In women, within‐visit CVs ranged from 5.0% to 35.0% (median = 13.0%), with slightly less variance in the VL compared to MH muscles. These CVs are somewhat higher than what has been reported in the calf/plantar flexion model across several labs (Adami & Rossiter, 2018; La Mantia et al., 2018; Southern et al., 2014; CV = 4.5%–11%) and markedly higher than the La Mantia et al. (2018) study (CV = 4.6%) which also used electrical stimulation in the resting VL of young adults. It is unclear why within‐visit (trial‐to‐trial) variability appears higher in the present study than in these previous reports (which were all in healthy young controls), particularly given the higher number of replicates we obtained (*n* = 4 vs. two or three replicates in previous reports) and our strict adherence to current NIRS testing guidelines (Adami & Rossiter, 2018; Barstow, 2019). It is possible that with a higher number of trials, there was a greater chance that one of the trials produced an abnormally high‐ or low‐rate constant value compared to the rest of the trials. While these occasional trials were not considered outliers based on *z*‐score analysis,they may still have increased the overall variability for that participant. Future studies should test whether the current protocol (i.e., bilateral stimulation and partial recovery curves) is inherently more variable than the standard unilateral 22 ischemic cuff protocol in these and other muscle groups. While somewhat higher than expected, the within‐visit variability observed with the present protocol is no higher than that reported for ^31^P‐NMR determination of PCr recovery kinetics (Ryan, Southern, et al., 2013) or muscle biopsy measures of mitochondrial capacity (Ryan, Brizendine, et al., 2013).

Between‐visit repeatability, a key determinant of the ability of any method to detect change over time or in response to intervention, was evaluated in all 16 subjects by repeating the same protocol on a second study visit at a similar time of day (experimental visits 1 and 2; individual data shown in Figure 5). In men, average *k* values for each limb on visit 2 were within 1% (VL muscle) and 5% (MH muscle) of the average values on visit 1, demonstrating good repeatability. Moreover, equivalency testing (Figure 6) showed that the mean difference was not different than zero (all *p* > 0.78) and the 90% confidence intervals were generally within 10% of the average rate constant for both muscles. Collectively these findings suggest that the bilateral NIRS protocol is repeatable across study visits in physically active younger men.

An unexpected finding, clearly shown in Figures 5 and 6C and D, was a significant between‐visit difference in *k* for both muscle groups in the women. We have no definitive explanation for the 10%–15% lower rate constants observed during the second visit in this group. The number of successful mitochondrial recovery curves (out of 16 total trials) was similar for the women compared to the men (Figure 4) suggesting that the overall quality of the data (i.e., monoexponential curve fits) was similar. However, closer inspection of individual data (data not shown) revealed that the three women with the longest time between visits (18, 31, and 32 days) also exhibited the largest between‐visit differences (−20.3, −20.4, −23.1%) in *k* in both of their VL muscles. Additionally, these 3 subjects had, in general, the highest average adipose tissue values in this study. While associations between ATT, time between studies, and variation (CV%) in the mitochondrial rate constant between visits were not found to be statistically significant (all *p* > 0.05, scatterplots not shown), these observations highlight the need for additional studies to identify determinants of low between‐visit repeatability of *k* with this protocol in these muscles.

### 4.3 Comparisons of *k* between muscle groups

The *k* values obtained in this study (group averages from 1.49 to 1.83 s^−1^) are consistent with values from the leg muscles of previous studies. For the VL muscle, Brizendine et al. (2013) and Erickson et al. (2013) reported average *k* values of 1.9 and 1.8, respectively, for moderately active subjects. Similar values for *k* have been reported in other studies as well (Sumner et al., 2020; Myers et al., 2019). For the medial gastrocnemius muscle, Lagerwaard et al. (2019) and Willingham et al. (2019) reported *k* values of 1.13 and 1.68 s^−1^, respectively, for inactive and moderately active subjects. While these prior studies used the full recovery curve (22–26 cuff occlusion) approach, the recent study by Sumner et al. (2020) reported that the six‐cuff approach (similar to that used in our study) provides equivalent *k* values.

To the best of our knowledge, there are no published NIRS‐based estimates of mitochondrial capacity for any of the hamstring muscles, likely due to the fact that these muscles are rarely biopsied and are generally viewed as less accessible for NIRS measurements than the quadriceps muscles. The hamstrings are an important muscle group to study because this is a frequently injured muscle group and a common site for tissue harvest in patients undergoing surgical ACL‐reconstruction. Our study showed that NIRS‐based estimates of mitochondrial capacity, while challenging in some subjects, could be acquired for this muscle group in subjects who met our study inclusion criteria (ATT  ≤2.0 cm, etc.).

We were surprised to find that average *k* values did not differ between the MH and VL, given reported differences in lab‐based endurance tests (Faxon et al., 2018) and EMG activity/motor unit recruitment during locomotor tasks (walking, running, jumping) between the quadriceps and hamstring muscle groups. We are not aware of any direct (biopsy‐based) or indirect (NIRS‐based or other methods) comparisons of mitochondrial capacity between the VL and hamstring muscles in humans. Additionally, muscle fiber type composition, a significant determinant of mitochondrial function, is quite variable across the few available hamstring biopsy studies in the literature (Evangelidis et al., 2017; Garrett et al., 1984; Johnson et al., 1973) and does not differ markedly (averaging 50%–60% type II fibers) from that reported for the VL (averaging 40%–60% type II fibers; Staron et al., 2000).

### 4.4 Comparisons of *k* between limbs

As seen in Figure 7, there was a moderately wide range (approximately two‐fold) of mitochondrial rate constants across subjects for each muscle. This variation may reflect the range of physical activity levels and our inclusion of both sexes. Despite this between‐subject variation in mitochondrial capacity, there was a strong agreement between limbs in each participant as indicated by high ICC values, low CVs. Bilateral consistency in NIRS‐based mitochondrial capacity was also suggested by the study of Harp et al. (2016) who reported no significant difference (*p* = 0.97) between recovery rate constants in the dominant versus non‐dominant calf muscles of healthy controls. That study also showed a trend for multiple sclerosis patients to have lower mitochondrial capacity in their weaker versus stronger leg (Harp et al., 2016). Taken together, these findings support the use of NIRS mitochondrial testing for comparing muscle oxidative capacity between limbs. The present protocol could be particularly useful in the serial evaluation of muscle‐ and limb‐specific pathology in unilateral diseases such as stroke, peripheral artery disease, or multiple sclerosis and in single limb exercise training studies designed to differentiate local versus systemic adaptive responses.

### 4.5 Comparisons of *k* between men and women

Although not a primary purpose of the present investigation, we did observe higher average mitochondrial rate constants in men than in women in both muscle groups and in both limbs (Figure 7). This sex difference was relatively small (~10% higher *k* values in men) and was directionally opposite of what would be expected based on these subjects' recent physical activity levels (lower in men) and predominant mode of training (eight of eight women participated in “endurance” activities vs. only two of eight men). This finding is also directionally opposite the differences in VL mitochondrial volume density (electron microscopy) and function (mitochondrial protein‐normalized oxidative enzyme activity in isolated muscle mitochondria) of moderately trained younger women and men matched for whole body aerobic capacity (Montero et al., 2018). These differences, however, should be viewed with caution due to their low effect sizes (Cohen's *d*: VL: 0.61; MH: 0.50) and the fact that this study was not powered (VL: *β* = 0.51; MH: *β* = 0.40) for sex comparisons. We estimate that 34 participants would be needed (using an effect size of 0.50) to determine with a power of 1 – *β* = 0.80 whether muscle mitochondrial capacity differs between active younger women and men based on an effect size of *d* = 0.61.

### 4.6 Study limitations

The primary purpose of this study was to develop, and test the reproducibility of, a novel bilateral NIRS mitochondrial capacity test in an extensively studied (VL) and never before reported (MH) lower‐limb muscle group. The sample we recruited was young, healthy, and physically active and included both sexes. It is highly representative of populations that have undergone the conventional NIRS mitochondrial test protocol in the published literature (i.e., 22+ cuff occlusion, single limb muscle) and reflects a population that is at highest risk for ACL injuries. Physical activity questionnaires are recognized as having shortcomings, including recall bias and over‐reporting of physical activity vs. more objective measures (e.g., accelerometry). However, the IPAQ questionnaire we used identified all 16 participants as either moderately or highly physically active, suggesting that none of our subjects were “sedentary” thereby facilitating comparisons of our reported mitochondrial rate constants with previously published cohorts. We did not strictly control physical activity between study visits, a factor that could have potentially contributed to the higher than expected between‐visit variability in the women's *k* values. Nevertheless, ANOVA revealed no significant effect of IPAQ score (s) as a covariate on the muscle *k* values we observed.

We did not compare the abbreviated (six cuff) method to the conventional (22–26 cuff) method in the present study. Thus, we cannot be certain that some of the variability we observed is a function of inadequate sampling or inherent within/across‐day variability. Such a comparison would require a much longer study visit (due to the need for adequate recovery between trials, etc.) and additional electrical stimulation and cuff inflations, but would have increased the external validity of our study given the 6‐cuff method has not been validated against ^31^P‐NMR.

Lastly, it is possible that variations in upper‐thigh cuff placement, slippage, timing, and pressure over repeated inflations contributed to the between‐visit variability in *k* values. However, we utilized Delfi cuffs (rigid and tapered) of sufficient width and an automated (LabView based) inflation‐deflation switch controller to minimize such variations. To the best of our knowledge, no one has systematically evaluated the influence of different cuff pressures on the resultant mitochondrial *k* values. However, such variations in cuff positioning, and so on are unlikely to have contributed in a major way to between‐visit variability in *k* values because when large differences in *k* were observed between the two study visits for a given subject, these differences were typically observed in both limbs.

### 4.7 Methodological considerations

The use of bilateral electrical stimulation was highly effective in controlling the contraction stimulus in both limbs simultaneously, but also presented a few challenges. First, due to the irregular shape and small external area of the MH muscle (relative to the VL), there was limited surface area available for the placement of two stimulation pads and the NIRS sensor. This was more frequently encountered with smaller subjects due to their shorter thigh lengths. However, this limitation did not appear to substantially reduce the quality of the monoexponential curve fitting or the repeatability of mitochondrial rate constants in the MH muscle (see Table 2). Second, the presence of more adipose tissue (i.e., higher ATT in the MH muscle and in women) also necessitated a higher current setting which led to the exclusion of six volunteers after initial screening. However, we ensured that the stimulation intensity was within the tolerance of each subject and all 16 subjects who completed this study adapted quickly to the transient stimulation of these muscles with minimal or no discomfort. Another issue was the deterioration of the adhesive stimulation pads after repeated trials, leading to a reduction in the conductance properties of the pads. This sometimes required a higher current setting to be applied (for a similar level of muscle activation) on experimental visit 2 compared to visit 1 for a given subject. Of note, a single set of stimulation pads was used for each subject and disposed after their final visit. The procedures described in our methods are recommended as a starting point for any investigators seeking to implement this protocol.

During pilot testing prior to this study, we set 1.5 cm as the exclusion criteria for both muscles, which is well below the estimated maximum penetration depth of the Portamon sensor (i.e., 2.0 cm). However, a few of our initial volunteers with MH ATT values slightly above this cut‐off (up to 1.65 cm) were found to exhibit an adequate (at least three‐fold) increase in metabolic rate during their initial electrical stimulation check (during volunteers' familiarization visit). From that point forward we used >1.5 and >1.7 cm as our ATT exclusion criteria for the VL and MH muscles, respectively. We do not know if the higher ATT cut‐off for the MH muscle, and thus the higher average adipose thickness of the MH versus VL muscle in our overall sample (Table 1), had an impact on the present results (most notably the poorer repeatability in a subset of our female participants). We also do not know for sure if switching from skinfold calipers to ultrasound imaging midway through our study increased the accuracy of our ATT estimates, although this is a reasonable assumption particularly in the thigh region (Selkow et al., 2011). Nonetheless, applying these ATT criteria with the Portamon device resulted in a high percentage of successful trials for these large muscle groups in most subjects (Figure 4).

Some investigators have recently suggested that the short duration muscle contractions used with the NIRS repeated cuff occlusion method (3 × 8 s in the present study) are not enough for mitochondria to be maximally activated and for muscle $\dot{V}O_{2}$ to reach a steady state (Zuccarelli et al., 2020). However, studies over the past ~25 years have shown that *k* values derived with this method do not appear to change with increasing muscle activation (intensity) or duration up to 5 min, similar to what is seen with the ^31^P‐NMR method. Thus, the use of short duration (to ensure oxygen does not become limiting), but sufficiently intense exercise (to rapidly activate mitochondrial enzymes) in repeated cuff occlusion protocols should not invalidate the “instantaneous” steady state reflected in the determination of *k*.

### 4.8 Conclusions and recommendations

The present study demonstrates the feasibility of NIRS‐based estimates of mitochondrial capacity in the VL and MH muscles using bilateral electrical stimulation and partial (six cuff) recovery curve analysis. This bilateral approach not only has the advantage of reducing testing time and subject discomfort and burden, but also yields a large number of recovery rate constants per muscle. This protocol demonstrated acceptable within‐visit repeatability in physically active young adults, but was susceptible to large between‐visit variability, particularly in women. This increased variability should be accounted for in future study designs.

It is recommended that investigators desiring to replicate this bilateral electrical stimulation protocol for future studies adhere (in general) to the standardized procedures described herein. Researchers are also strongly encouraged, as highlighted in the recent CORP review in this journal (Barstow, 2019), to follow the precautions described above with respect to adipose tissue measurements and exclusion criteria to improve the accuracy and reproducibility of the derived *k* values for their particular muscle group(s) and population(s) of interest. Investigators wishing to track changes in mitochondrial capacity over time and/or to document the effects of an intervention should closely monitor concurrent changes in weekly (possibly limb‐specific) physical activity and employ other relevant methodological controls (changes in health status/weight, documentation of menstrual cycle phase during testing (Stanhewicz & Wong, 2020). Lastly, the data quality, group means, and individual variability provided in this report provides novel reference data for future studies seeking to improve upon the quality and reproducibility of NIRS‐based estimates of muscle mitochondrial capacity.

## DISCLOSURES

The authors declared no potential conflicts of interest with respect to the research, authorship and/or publication of this article.

## AUTHOR CONTRIBUTIONS

Conception and design: Rewais Hanna, Jigar Gosalia, Zachary Hobson, Kevin K. McCully, Brian A. Irving, Swapan Mookerjee, Giampietro L. Vairo, and David N. Proctor; perform experiments: Rewais Hanna, Jigar Gosalia, Alaina Demalis; analyze data: Rewais Hanna, Jigar Gosalia, and David N. Proctor; interpret results: Rewais Hanna, Jigar Gosalia, Kevin K. McCully, Brian A. Irving, Swapan Mookerjee, Giampietro L. Vairo, and David N. Proctor; prepare figures: Rewais Hanna, Jigar Gosalia, and David N. Proctor; draft manuscript: Rewais Hanna, Jigar Gosalia, Giampietro L. Vairo, and David N. Proctor; edit/revise and approve manuscript: all authors.

## Data Availability

The data that support the findings of this study are available from the corresponding author upon reasonable request.
